# Supplementary material for: Genomic divergence during feralization reveals both conserved and distinct mechanisms of parallel weediness evolution
Source: Commun Biol. 2021 Aug 10;4:952. doi: 10.1038/s42003-021-02484-5 (PMC8355325; doi:10.1038/s42003-021-02484-5)
Supplement: Supplementary file 2 — Supplementary Information [file 42003_2021_2484_MOESM2_ESM.pdf]

Supplementary Information

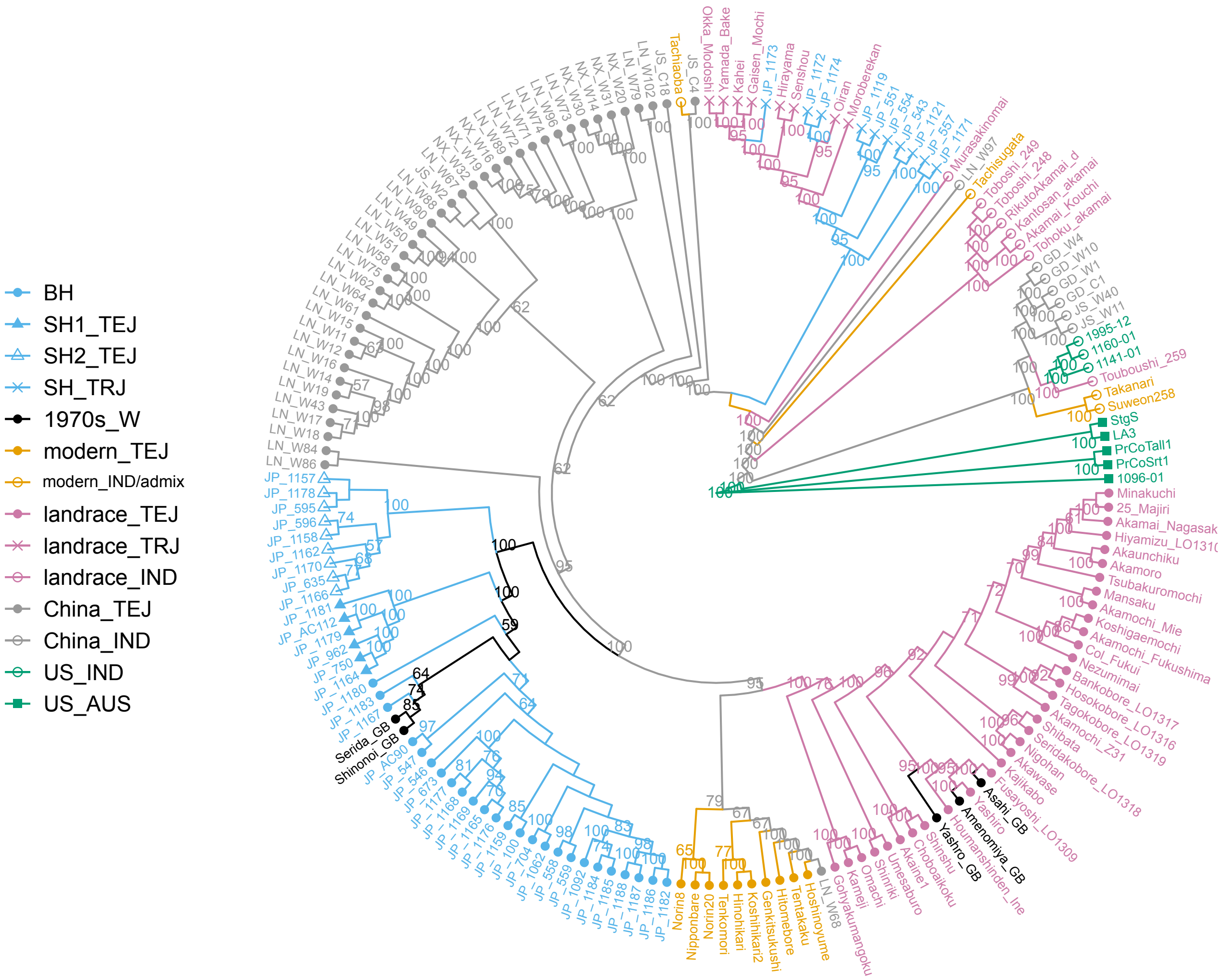

Supplementary Fig. 1. Phylogenetic tree of 174 rice strains including weedy and cultivated rice as shown in Fig. 1e, but with strain names. Different sample groups are coded with different colours: light blue for Japanese weedy rice; black for 1970s_W; orange for modern cultivated rice, pink for landrace cultivated rice; gray for Chinese weedy rice; green for US weedy rice. Bootstrap values (>50) are shown on each branch.

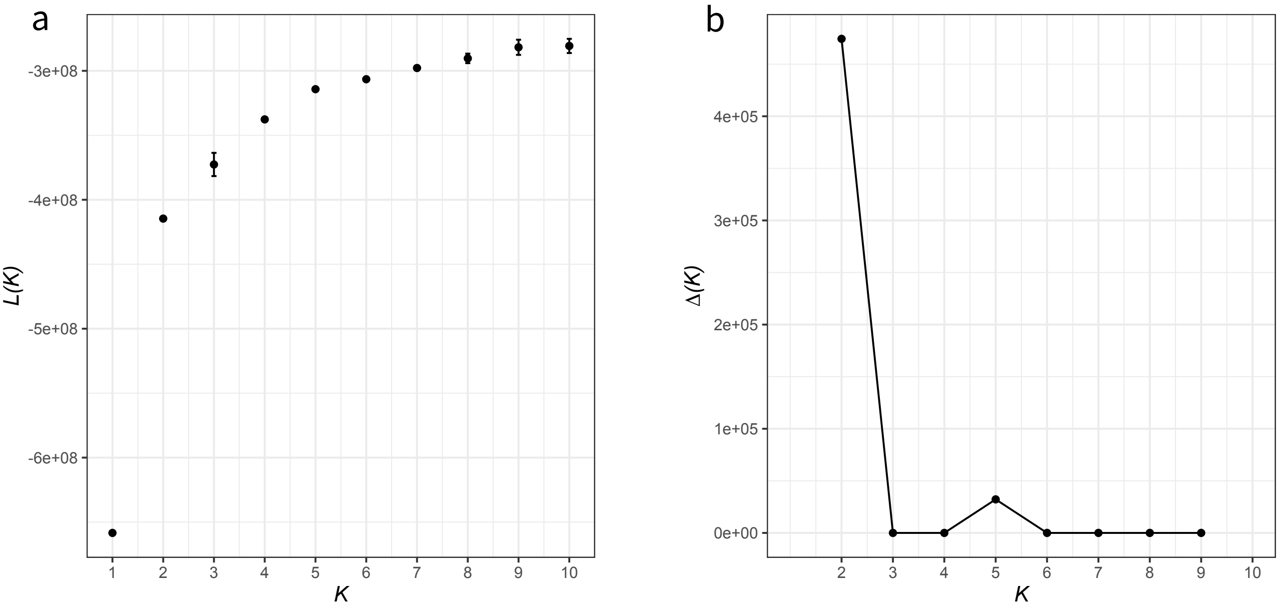

Supplementary Fig. 2. Optimal number of *K* value evaluated by *L(K)* **and Δ*K* values. a.** Mean *L(K)*) (±SD) over 10 runs for each *K* value up to *K* = 10. **b.** Δ*K* values for each *K* value up to *K* = 9.

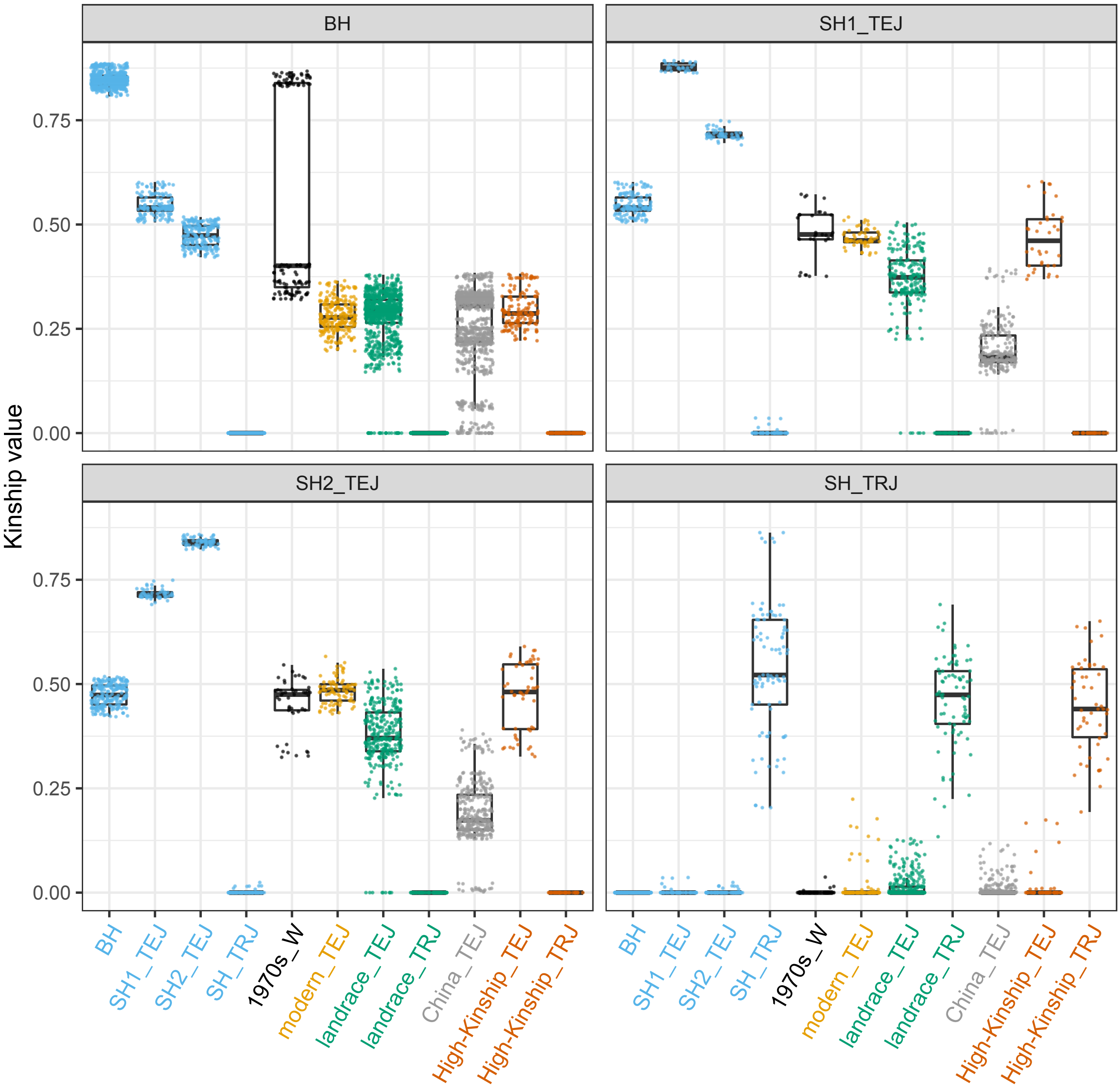

Supplementary Fig. 3. Boxplot of Kinship values between each group and BH (topleft), SH1_TEJ (topright), SH2_TEJ (bottomleft), and SH_TRJ (bottomright). Each point indicates individual Kinship values between two strains. High-Kinship_TEJ and _TRJ are cultivated rice strains that have been shown to have high Kinship values with Japanese weedy rice strains according to Qiu et al. (2020). The detailed information for High-Kinship_TEJ and _TRJ is described in Supplementary Table 1. Each group consists of multiple strains (biologically independent samples): n=25 in BH; n=6 in SH1_TEJ; n=9 in SH2_TEJ; n=10 in SH_TRJ; n=5 in 1970s_W; n=10 in modern_TEJ; n=33 in landrace_TEJ; n=8 in landrace_TRJ; n=41 in China_TEJ; n=7 in High-Kinship_TEJ; n=4 in High-Kinship_TRJ.

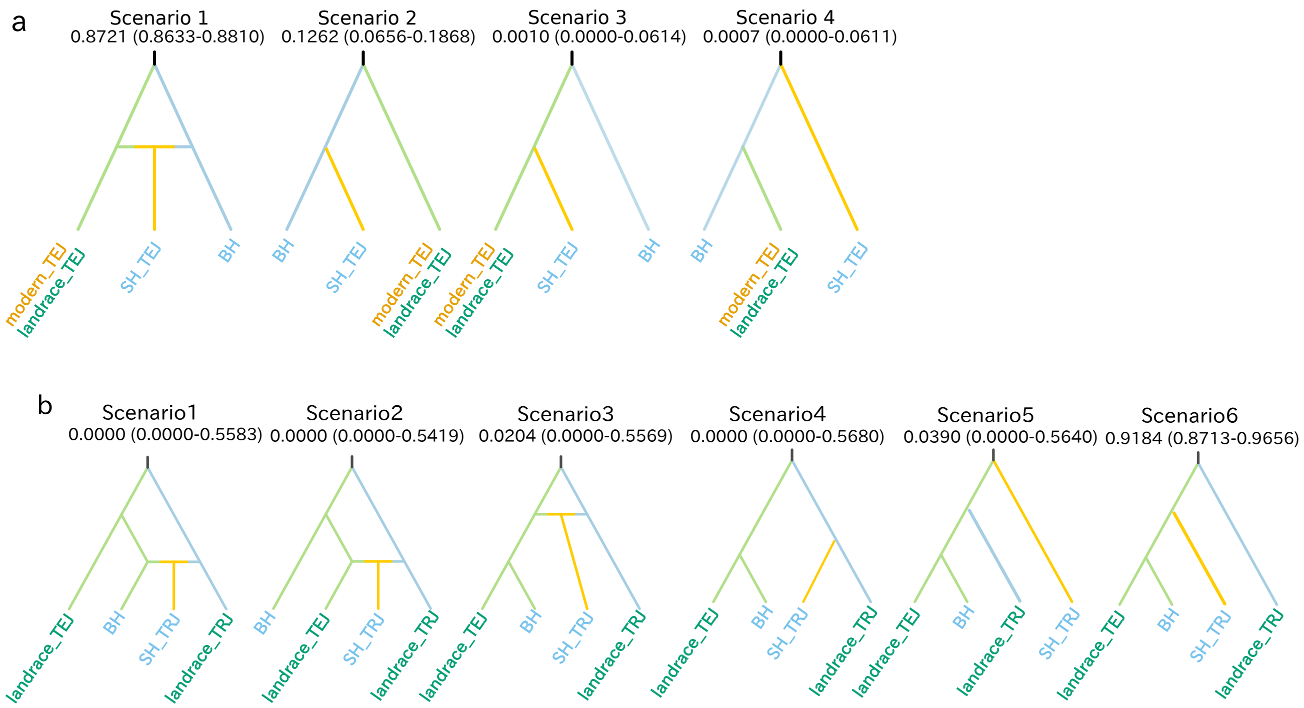

Supplementary Fig. 4. Demographic scenario models for the evolution of weedy rice strains.
a. Models for the evolution of BH and SH_TEJ weedy rice. Scenario 1: BH weedy rice and Japanese cultivated rice evolved from a shared ancestor, then SH_TEJ weedy rice evolved from a hybridization event between BH weedy and cultivated rice; Scenario 2: Japanese cultivated rice and shared weedy rice ancestor of BH and SH_TEJ diverged at first, then BH and SH_TEJ weedy rice diverged from the shared ancestor; Scenario 3: BH weedy rice diverged from a shared ancestor earlier than SH_TEJ weedy rice; and Scenario 4: SH_TEJ weedy rice diverged from a shared ancestor earlier than BH weedy rice. Posterior Probability (95% of confidence interval) for each scenario was indicated under the title of each scenario.
b. Models for the evolution of SH_TRJ weedy rice. Scenario 1: SH_TRJ weedy rice evolved from a hybridization event between landrace_TRJ and BH weedy rice; Scenario 2: SH_TRJ weedy rice evolved from a hybridization event between landrace_TRJ and landrace_TEJ; Scenario 3: SH_TRJ weedy rice evolved from a hybridization event between landrace_TRJ and TEJ strains before the divergence of landrace_TRJ and BH weedy rice; Scenario 4: TRJ and TEJ strains diverged from a shared ancestor, after which SH_TRJ weedy rice diverged from landrace_TRJ; Scenario 5: SH_TRJ weedy rice diverged from a shared ancestor before the divergence of landrace_TRJ and TEJ strains; Scenario 6: landrace_TRJ diverged from a shared ancestor before the divergence of SH_TRJ weedy rice and TEJ strains. Posterior Probability (95% of confidence interval) for each scenario was indicated under the title of each scenario.

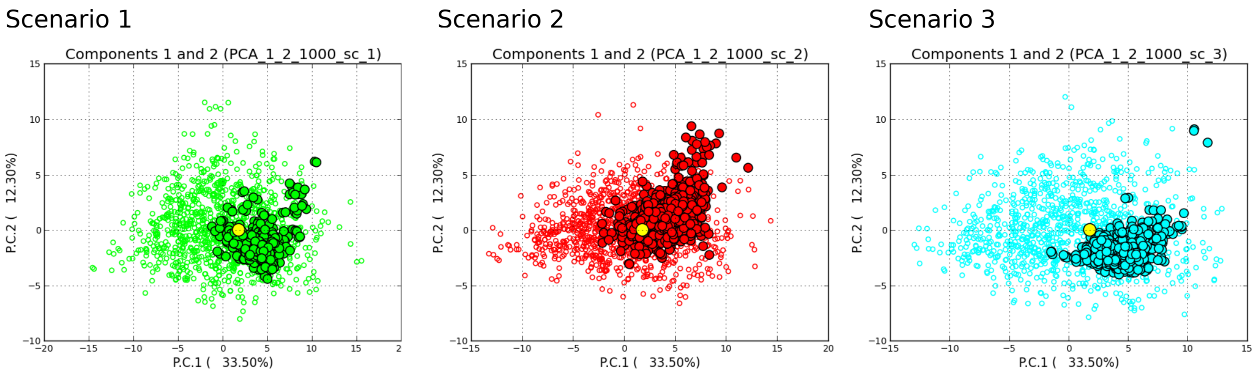

Supplementary Fig. 5. Model checking for the three demographic scenarios shown in Fig. 2b. Results are shown on the PCA plots. Open and filled circles represents the simulated datasets of priors and posterior, respectively, and the large yellow circle represents the observed datasets. If the model fit is acceptable, the observed data (large yellow circle) is observed within the scope of simulated posterior datasets (filled circle).

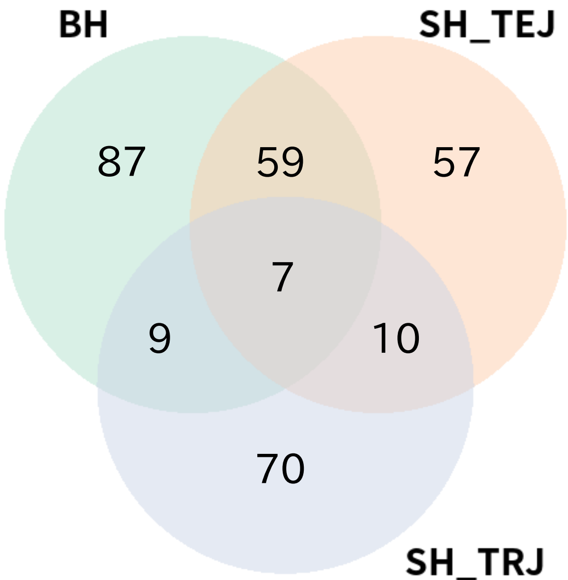

Supplementary Fig. 6. Venn diagram showing the number of outliers of the F_{ST} (at least the five deviations away from the median) between weedy rice groups.

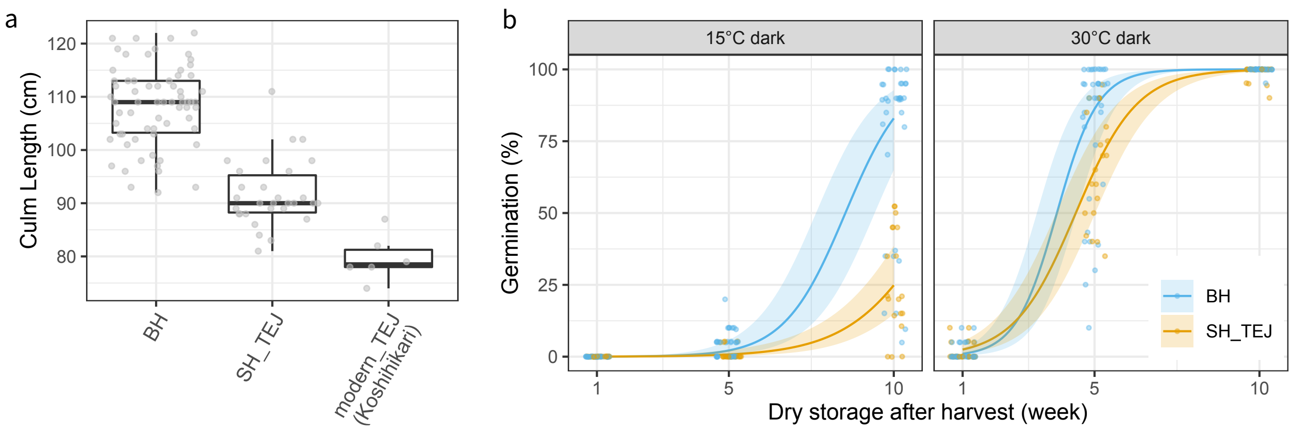

Supplementary Fig. 7. Differences of culm length and seed dormancy between BH and SH_TEJ weedy rice.

a. Culm length were evaluated during heading for n=6 biologically independent samples per stain and each point indicates culm length of each individual. BH weedy rice (n=11 strains (biologically independent samples)), SH_TEJ weedy rice (n=5 strains) and one modern_TEJ cultivated rice (Koshihikari) were used to compare culm length among subgroups.

b. Germination after different periods of dry storage. Germination rates were estimated by generalized liner mixed model with binomial link and strains were used as a random effect. Shaded areas represent a 95% confidence interval band for each curve and each point indicates germination rates for each replicate for each strain and each time point. Seeds of BH (n=8 strains) and SH_TEJ (n=5 strains) weedy rice were collected from farmer's paddy rice fields in 2016.

BH weedy rice: JP_1165, JP_1167, JP_1168, JP_1169, JP_1177, JP_1180, JP_1182, JP_1183, JP_1184, JP_1185, JP_1187 (JP_1168, JP_1184 and JP_1187 were not used for evaluation of seed dormancy); SH_TEJ weedy rice: JP_1166, JP_1170, JP_1178, JP_1179, JP_1181.

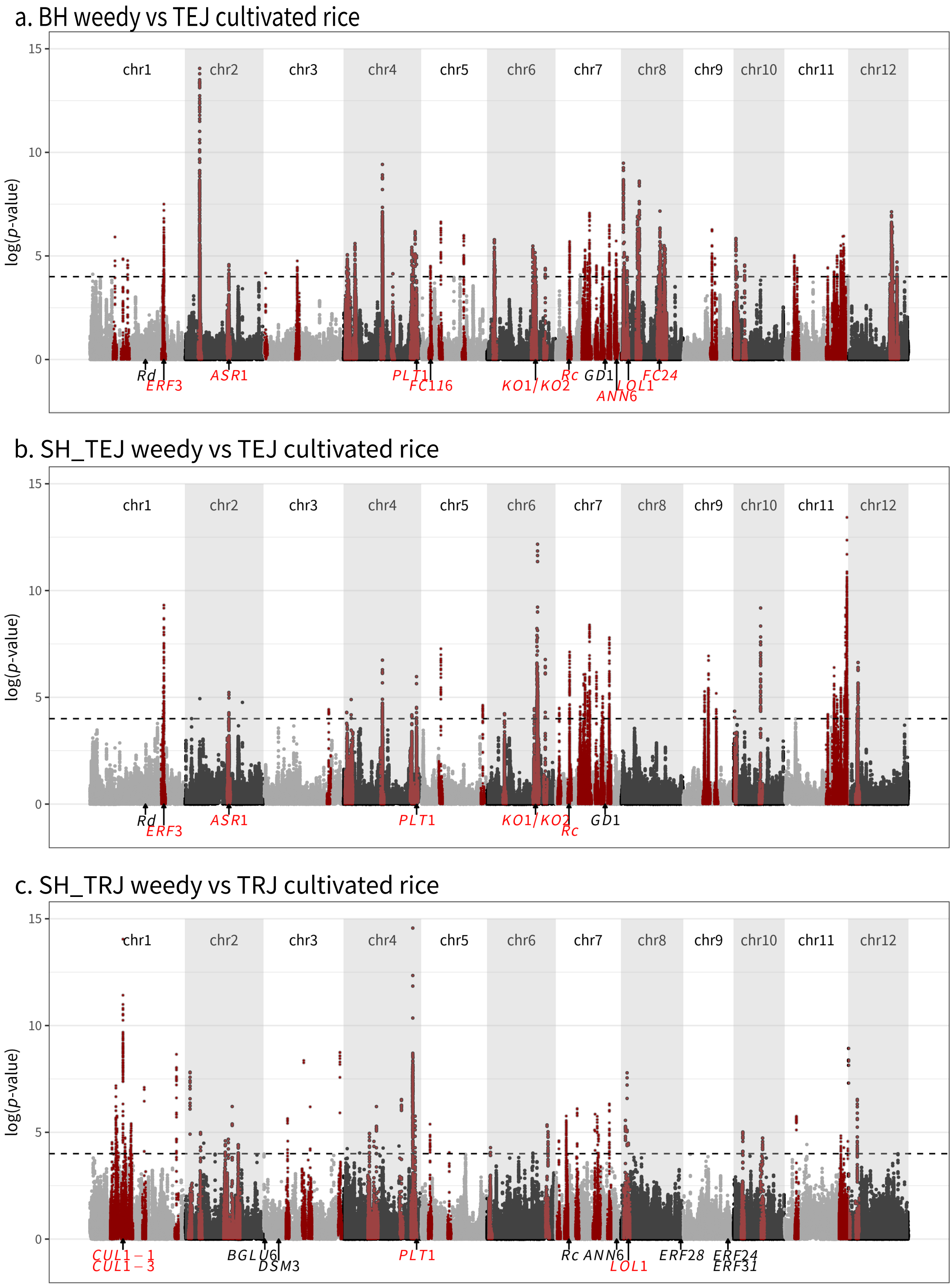

Supplementary Fig. 8. Plot of XP-EHH between TEJ cultivated rice and BH and SH_TEJ weedy rice (a and b, respectively) and between TRJ cultivated and SH_TRJ weedy rice (c). The associated *p*-value are indicated instead of the XP-EHH score itself. Candidate genes that were included in the regions under positive selection by F_{ST} (Fig. 3) were described and genes that were included in the regions under positive selection by XP-EHH tests were shown in dark red.

Supplementary Table 1. Choloroplast genome haplotypes in TEJ/TRJ cultivated rice and TEJ/TRJ-derived weedy rice.

variant position	REF	ALT	variants for each haplotype						
			Haplotype 1	Haplotype 2	Haplotype 3	Haplotype 4	Haplotype 5	Haplotype 6	Haplotype 7
17379	TATAGAA	T	REF	ALT	REF	REF	ALT	REF	ALT
26653	A	G	REF	ALT	REF	REF	REF	REF	REF
57026	T	A	REF	REF	REF	REF	missing	REF	REF
57033	ACTTTTTTTTTTAGAATACTAAAGTATTCTAAAAAAAAAAGTATTCTATAAAAAAT	A	REF	REF	REF	ALT	missing	ALT	REF
57052	AAAGTATTCTAAAAAAAAAAGTATTCTATAAAAAAT	TAAGTATTCTAAAAAAAAAAGTATTCTATAAAAAAT	REF	REF	ALT	missing	REF	missing	REF
57053	AAG	TAG	REF	REF	ALT	REF	REF	missing	REF
57061	TAAA	TAA	REF	ALT	REF	REF	ALT	missing	ALT
57079	T	A	REF	ALT	REF	REF	REF	missing	ALT

Supplementary Table 2. Nonsynonymous variants in candidate gense.

gene name	variant	weedy rice			cultivated rice		
		BH (25 strains)	SH_TEJ (15 strains)	SH_TRJ (10 strains)	modern_TEJ (10 strains)	landrace_TEJ (33 strains)	landrace_TRJ (8 strains)
FC116	c.487G>T<br>p.Ala163Ser	ALT (21 strains)<br>Het (4)	REF (15)	REF (9)<br>Het (1)	REF (10)	REF (33)	REF (8)
KO1	c.70G>A<br>p.Ala24Thr	ALT(24)<br>Het (1)	REF (15)	ALT (9)<br>Het (1)	REF (2)<br>ALT (7)<br>Empty (1)	REF (8)<br>ALT (23)<br>Het (1)<br>Empty (1)	REF (1)<br>ALT (6)<br>Empty (1)
	c.1098G>C<br>p.Gln366His	ALT (19)<br>Het (6)	REF (15)	ALT (9)<br>Het (1)	REF (2)<br>ALT (8)	REF (10)<br>ALT (20)<br>Het (3)	REF (1)<br>ALT (6)<br>Empty (1)
	c.1163T>A<br>p.Met388Lys	ALT (20)<br>Het (5)	REF (15)	ALT (7)<br>Het (3)	REF (2)<br>ALT (8)	REF (10)<br>ALT (21)<br>Het (2)	ALT (8)
	c.1364C>T<br>p.Thr455Ile	ALT (19)<br>Het (6)	REF (15)	ALT (9)<br>Het (1)	REF (2)<br>ALT (8)	REF (10)<br>ALT (19)<br>Het (4)	REF (1)<br>ALT (6)<br>Empty (1)
KO2	c.467T>C<br>p.Ile156Thr	ALT(24)<br>Het (1)	REF (15)	ALT (9)<br>Het (1)	REF (2)<br>ALT (8)	REF (8)<br>ALT (23)<br>Het (2)	ALT (8)
	c.1367C>T<br>p.Thr456Ile	ALT (24)<br>Het (1)	REF (12)<br>Het (3)	ALT (9)<br>Het (1)	REF (2)<br>ALT (7)<br>Het (1)	REF (7)<br>ALT (24)<br>Het (2)	ALT (6)<br>Het (2)
GD1	c.196_204dupCAGCAGCAG<br>p.Gln66_Gln68dup	ALT(24)<br>REF (1)	ALT(14)<br>REF (1)	REF (6)<br>ALT (3)<br>Het (1)	REF (9)<br>Empty (1)	REF (29)<br>Empty (4)	REF (2)<br>ALT (6)
LOL1	c.607C>T<br>p.Gln203*	REF (25)	REF (15)	REF (10)	REF (9)	REF (27)<br>ALT (7)	REF (4)<br>ALT (4)
