# Supplementary material for: Genomic divergence during feralization reveals both conserved and distinct mechanisms of parallel weediness evolution
Source: Commun Biol. 2021 Aug 10;4:952. doi: 10.1038/s42003-021-02484-5 (PMC8355325; doi:10.1038/s42003-021-02484-5)
Supplement: Supplementary file 3 — Description of Additional Supplementary Files [file 42003_2021_2484_MOESM3_ESM.pdf]

Description of Additional Supplementary Files

File name: Supplementary Data 1

Description: The list of weedy and cultivated rice strains used in this study.

File name: Supplementary Data 2

Description: Ancestry proportions for weedy and cultivated rice strains.

File name: Supplementary Data 3

Description: Variations in candidate genes, *FC116, KO1, KO2, GD1* and *LOL1.*
