# Supplementary material for: Genomic divergence during feralization reveals both conserved and distinct mechanisms of parallel weediness evolution
Source: Commun Biol. 2021 Aug 10;4:952. doi: 10.1038/s42003-021-02484-5 (PMC8355325; doi:10.1038/s42003-021-02484-5)
Supplement: Supplementary file 7 — Reporting Summary [file 42003_2021_2484_MOESM7_ESM.pdf]

nature research

Corresponding author(s): Toshiyuki Imaizumi
Last updated by author(s): Jun 21, 2021

Reporting Summary

Nature Research wishes to improve the reproducibility of the work that we publish. This form provides structure for consistency and transparency in reporting. For further information on Nature Research policies, see our Editorial Policies and the Editorial Policy Checklist.

Statistics

For all statistical analyses, confirm that the following items are present in the figure legend, table legend, main text, or Methods section.

n/a	Confirmed	
☐	☒	The exact sample size (*n*) for each experimental group/condition, given as a discrete number and unit of measurement
☐	☒	A statement on whether measurements were taken from distinct samples or whether the same sample was measured repeatedly
☐	☒	The statistical test(s) used AND whether they are one- or two-sided<br>*Only common tests should be described solely by name; describe more complex techniques in the Methods section.*
☒	☐	A description of all covariates tested
☐	☒	A description of any assumptions or corrections, such as tests of normality and adjustment for multiple comparisons
☐	☒	A full description of the statistical parameters including central tendency (e.g. means) or other basic estimates (e.g. regression coefficient) AND variation (e.g. standard deviation) or associated estimates of uncertainty (e.g. confidence intervals)
☐	☒	For null hypothesis testing, the test statistic (e.g. *F*, *t*, *r*) with confidence intervals, effect sizes, degrees of freedom and *P* value noted<br>*Give P values as exact values whenever suitable.*
☐	☒	For Bayesian analysis, information on the choice of priors and Markov chain Monte Carlo settings
☐	☒	For hierarchical and complex designs, identification of the appropriate level for tests and full reporting of outcomes
☒	☐	Estimates of effect sizes (e.g. Cohen's *d*, Pearson's *r*), indicating how they were calculated

Our web collection on statistics for biologists contains articles on many of the points above.

Software and code

Policy information about availability of computer code

Data collection	SRA-Toolkit version 2.8.2
Data analysis	Trimmomatic version 0.38, BWA version 0.7.17, Picard tools version 2.18.17, GATK version 4.0.11.0, Beagle version 5.0, MEGA7, R 3.6.3, ggtree version 1.1.6, PCAngsd version 0.97, NGSadmix, NGSrelate, ANGSD, VCFtools, rehh version 3.1.2, Loter, DIYABC version 2.1.0. All software and codes used in this study are publicly available.

For manuscripts utilizing custom algorithms or software that are central to the research but not yet described in published literature, software must be made available to editors and reviewers. We strongly encourage code deposition in a community repository (e.g. GitHub). See the Nature Research guidelines for submitting code & software for further information.

Data

Policy information about availability of data

All manuscripts must include a data availability statement. This statement should provide the following information, where applicable:

- Accession codes, unique identifiers, or web links for publicly available datasets
- A list of figures that have associated raw data
- A description of any restrictions on data availability

All whole genome sequence data are available in SRA/ENA/DRA (DRR209178-DRR209229 and DRR255840-DRR258879).

Field-specific reporting

Please select the one below that is the best fit for your research. If you are not sure, read the appropriate sections before making your selection.

☐ Life sciences ☐ Behavioural & social sciences ☒ Ecological, evolutionary & environmental sciences

For a reference copy of the document with all sections, see nature.com/documents/nr-reporting-summary-flat.pdf

Ecological, evolutionary & environmental sciences study design

All studies must disclose on these points even when the disclosure is negative.

Study description	We analysed whole-genome sequences of weedy and cultivated rice in Japan to characterize the genomic basis of evolution in weedy rice.
Research sample	The research sample constituted of 50 contemporary Japanese weedy rice strains, five weedy rice strains collected in the 1970s, 33 Japanese landraces, and published whole genome sequences for 86 cultivated and weedy rice strains.
Sampling strategy	No statistical methods were used to predetermine sampling strategy.
Data collection	Contemporary weedy rice strains were collected from farmer's fields, and seeds for all strains of the 1970s weedy rice and landraces were obtained from the Genetic Resources Center or Central Region of Agricultural Research Center, National Agricultural and Food Research Organization (NARO), Japan or Institute of Genetic Resources, Faculty of Agriculture, Kyushu University, Japan.
Timing and spatial scale	Contemporary weedy rice strains were collected from 2014 to 2017.
Data exclusions	For the whole-genome sequencing analysis, the SNP calls were filtered according to the following thresholds ‘QD < 5.0, FS > 50.0, SOR > 3.0, MQ < 50.0, MQRankSum < -2.5, ReadPosRankSum < -1.0, ReadPosRankSum > 3.5’ to reduce false positives. Imputation of all reported SNPs in the 174 strains from variant call format (VCF) was performed by Beagle v5.0 with default parameters using the genotype likelihoods. To investigate demographic history of weedy rice, all analyses were based on a subset of 4,289 SNPs extracted from 212,319 homozygous SNPs among Japanese cultivated and weedy rice using VCFtools with 10,000 thinned methods (extracting one SNP per 10,000 bp).
Reproducibility	For phenotypic classification, we evaluated culm length and seed dormancy. Culm length was evaluated during heading for 6 individuals per strain that were grown in an experimental field, and 11 black hull and 5 straw hull weedy rice strains collected from different populations were used to compare culm length among weedy rice groups. For the evaluation of seed dormancy, germination assays were conducted on seeds collected from different farmers’ fields for 8 black hull and 5 straw hull weedy rice strains in a factorial design (13 weedy rice strains x 2 temperature x 3 after-ripening x 4 replicates).
Randomization	Plant growth of weedy rice for evaluating culm length was in a randomized block design and seeds used in germination assays were randomly collected from multiple individuals from farmers' fields.
Blinding	Blind was not relevant to this study, which is standard in the population genetics field.

Did the study involve field work? ☐ Yes ☒ No

Reporting for specific materials, systems and methods

We require information from authors about some types of materials, experimental systems and methods used in many studies. Here, indicate whether each material, system or method listed is relevant to your study. If you are not sure if a list item applies to your research, read the appropriate section before selecting a response.

Materials & experimental systems

n/a	Involved in the study	
☒	☐	Antibodies
☒	☐	Eukaryotic cell lines
☒	☐	Palaeontology and archaeology
☒	☐	Animals and other organisms
☒	☐	Human research participants
☒	☐	Clinical data
☒	☐	Dual use research of concern

Methods

n/a	Involved in the study	
☒	☐	ChIP-seq
☒	☐	Flow cytometry
☒	☐	MRI-based neuroimaging
